# Evaluation of factors affecting external tibial torsion in patients with cerebral palsy

**DOI:** 10.1186/s12891-021-04570-5

**Published:** 2021-08-12

**Authors:** Jae Jung Min, Soon-Sun Kwon, Kyu Tae Kim, Young Choi, Ki Hyuk Sung, Kyoung Min Lee, Moon Seok Park

**Affiliations:** 1 Department of Orthopaedic Surgery, Seoul National University Bundang Hospital, Seongnam-si,, Gyeonggi South Korea; 2 Department of Mathematics, College of Natural Sciences, Ajou University, Suwon-si, Gyeonggi South Korea; 3 Department of Orthopaedic Surgery, Kosin University Gospel Hospital, Pusan, South Korea

**Keywords:** Cerebral palsy, Femoral anteversion, Gait deviation, Hemiplegia, Tibial torsion

## Abstract

**Background:**

Gait deviation and associated torsional problems are common in patients with cerebral palsy (CP). Although femoral anteversion in CP has been extensively reviewed in previous studies, only a few studies have focused on tibial torsion. Therefore, this study aimed to evaluate tibial torsion in patients with CP and investigate the affecting factors.

**Methods:**

Consecutive patients with cerebral palsy who underwent 3-dimensional computed tomography for the assessment of rotational profiles were reviewed. Femoral anteversion and tibial torsion were measured, and the demographic characteristics of the patients were recorded. A linear mixed model was implemented to overcome the retrospective nature of the study.

**Results:**

After the implementation of inclusion and exclusion criteria, 472 patients were enrolled for this study. With age, external tibial torsion increased, while femoral anteversion decreased. The factors affecting external tibial torsion were increased femoral anteversion (*p* = 0.0057), increased age (*p* < 0.0001), higher Gross Motor Function Classification System (GMFCS) level (*p* < 0.0001), and involved/uninvolved limbs of hemiplegia (*p* = 0.0471/*p* = 0.0047).

**Conclusions:**

Older age, GMFCS level IV/V, hemiplegia, and increased femoral anteversion were the independent risk factors of increased **external** tibial torsion; therefore, performing an imaging study is recommended for assessing the extent of tibial torsion in patients with such characteristics.

## Background

Gait deviation in the transverse plane is common in patients with cerebral palsy (CP). It comprises lever arm dysfunctions, resulting in inefficient energy consumption and problems in gait appearance [1]. Increased femoral anteversion, external tibial torsion, abnormal foot appearance, and muscle imbalance attribute to gait problems in the transverse plane [2]. Most of all, increased femoral anteversion is notable in patients with CP. Femoral anteversion in CP patients increases according to the Gross Motor Function Classification System (GMFCS) level [3], and it is believed to not improve with age. Therefore, femoral derotational osteotomy is one of the common procedures used in single-event multilevel surgery to improve gait function in patients with CP [4–6].

Although femoral anteversion in CP has been extensively reviewed in previous studies, only a few studies focused on **external** tibial torsion. This lack of research is owing to the paucity of pathologic tibial torsion compared with increased femoral anteversion, and physical examination depicting tibial torsion is not as good as that depicting femoral anteversion in terms of validity [7, 8]. With the above background, this study aimed to evaluate **external** tibial torsion in patients with CP and investigate its affecting factors, such as age, GMFCS level, and concomitant deformities.

## Methods

### Ethical statements

This study was approved by the institutional review board (IRB) of our hospital (a tertiary referral center of CP, IRB number: B-2003-601-103), and it was performed in accordance with the guidelines of the World Medical Association Declaration of Helsinki. The need of obtaining informed consent was waived because of the retrospective nature of this study.

### Participants

**Fig. 1:**
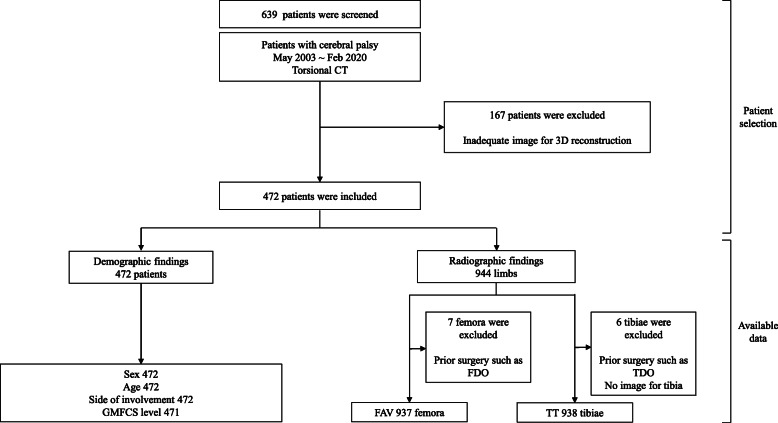
Inclusion and exclusion criteria for patients in this study3D, three-dimensional; CT, computed tomography; FAV, femoral anteversion; FDO, femoral derotational osteotomy; GMFCS, Gross Motor Function Classification System; TDO, tibial derotational osteotomy; TT, tibial torsion

Overall, 639 patients were screened using a clinical data warehouse (CDW) in our hospital [Healthcare Information and Management Systems Society (HIMSS), stage 7] according to the following inclusion criteria: (1) consecutive patients with CP between March 2003 and December 2019, (2) patients aged < 18 years at the time of assessment, and (3) patients who had torsional 3-dimensional (3D) computed tomography (CT) scans. The exclusion criteria were as follows: (1) inadequate 3D CT scan for measuring femoral anteversion or tibial torsion, (2) patients with a history of orthopedic intervention (bony or soft-tissue procedures) for the treatment of CP before assessment, and (3) patients with neuromuscular diseases other than CP (Fig. 1).

### Data collection

After implementing the inclusion and exclusion criteria, two authors (MJJ and PMS) reviewed the patients’ medical records. Patients’ age at surgery, sex, GMFCS level, involvement (unilateral/bilateral), and date of the 3D CT scan were included as demographic data.

### Building consensus and reliability

Five authors (JJM, KHS, KML, and MSP, who are orthopedic surgeons with 3, 17, 18, and 20 years of experience, respectively, and S-SK, a statistician) held a consensus-building session for the CT measurements and agreed on the methods of the measurements. Previous studies on CT measurements were reviewed, and 3D images were used for measurements. Before the main measurement, three authors (JJM, KHS, and MSP) measured femoral anteversion and tibial torsion to ensure interobserver reliability, and one of the authors (JJM) performed the measurements after 4 weeks to ensure interobserver reliability.

CT images (Mx8000-IDT; Philips Healthcare Korea, Seoul, South Korea) were used in this study. Femoral anteversion and tibial torsion were measured using the picture archiving and communication system software (INFINITT Healthcare, Seoul, Korea), and the Rapidia software (version 2.8; INFINITT Healthcare) reconstructed the 3D image from the CT scan. Following the reliability testing, two authors (MJJ and PMS) measured the CT indices.

### Definitions

**Fig. 2:**
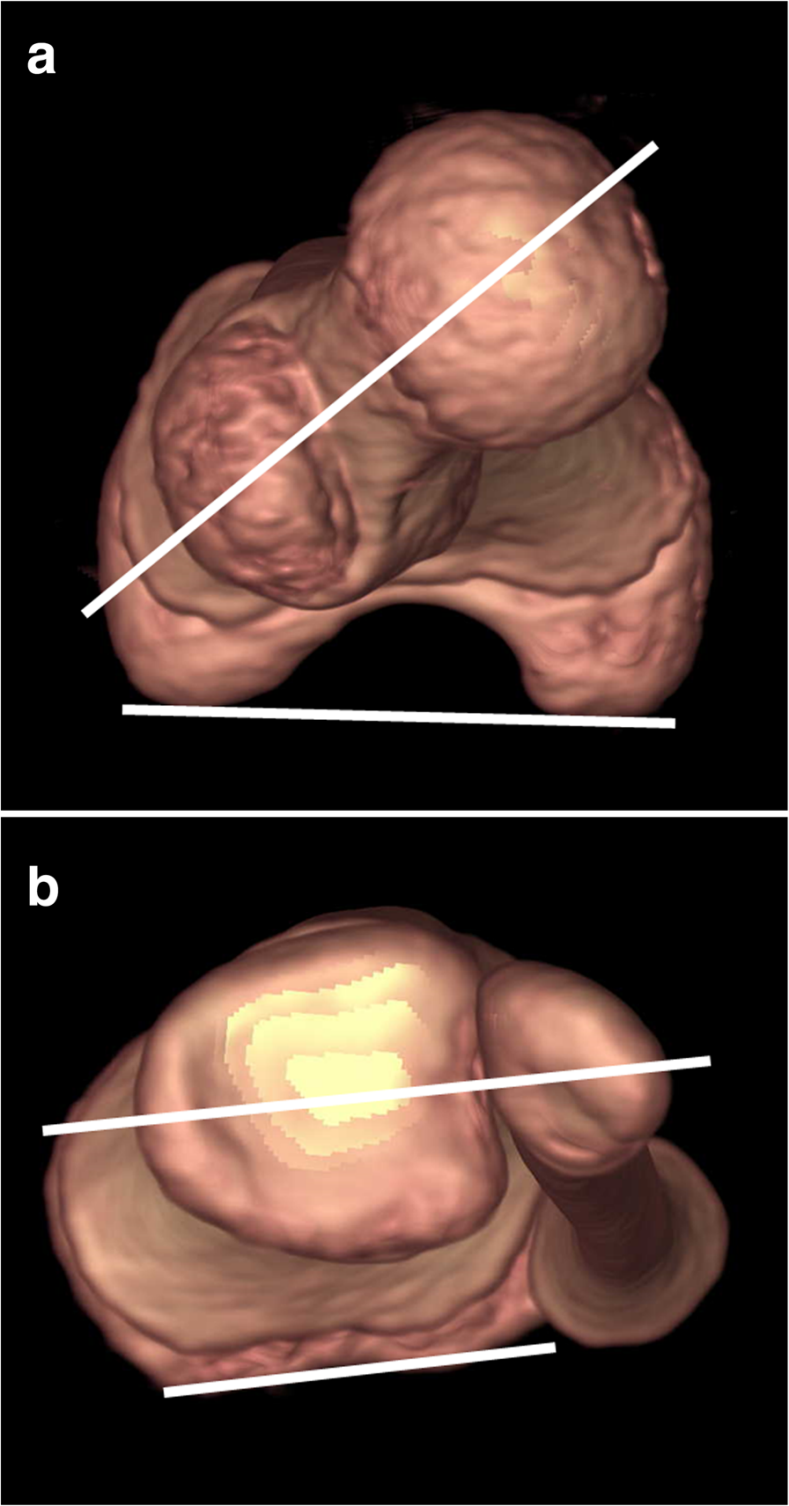
Axial three-dimensional computed tomography scans (**A**) Femoral anteversion is the angle between the line connecting the centers of the femoral head and greater trochanter and another line connecting the posterior margins of the medial and lateral femoral condyles. (**B**) Tibial torsion is the angle between the line connecting the posterior margins of the medial and lateral tibial condyles and another line connecting the midpoints of the medial malleolus and syndesmotic articular surface of the lateral malleolus

On an axial 3D CT scan, femoral anteversion was defined as the angle between a line connecting the centers of the femoral head and greater trochanter and another line connecting the posterior margins of the medial and lateral femoral condyles (Fig. 2 A). Tibial torsion was defined as the angle between a line connecting the posterior margins of the medial and lateral tibial condyles and another line connecting the midpoints of the medial malleolus and syndesmotic articular surface of the lateral malleolus (Fig. 2 B).

### Building a linear mixed model

Tibial torsion was adjusted by multiple factors using a linear mixed model, with sex, GMFCS level, involvement (bilateral/unilateral), and ipsilateral femoral anteversion as the fixed effects, and laterality and each subject as the random effects. The covariance structure was assumed as the variance components. The restricted maximum likelihood estimation was used to estimate parameters for the linear mixed model [9, 10]. A linear mixed model with a random slope and a random intercept was suggested. The slope was tibial torsion according to age. The models were accepted as valid for estimating the responses according to the Akaike information criterion (AIC) and Bayesian information criterion (BIC). A smaller AIC or BIC value is preferred in terms of model selection.

### Statistical analysis

Descriptive statistics was used to summarize the patients’ demographics and CT measurements. Data normality was determined using the Kolmogorov-Smirnov test.

This study used intraclass correlation coefficients (ICCs) for reliability testing [11, 12]. The required sample size for reliability testing of CT indices was calculated before the reliability session. The target value of ICCs for CT and radiographic measurements was 0.8 with a 95% confidence interval (CI) of 0.2. The sample size was calculated using the Bonnet approximation [13] (36 limbs for two observers). Eighteen right legs and 18 left legs were randomly selected for statistical independence and included for reliability testing [13]. ICCs and their 95% CIs were determined in the setting of a two-way random effect model, a single measurement, and absolute agreement [11, 12].

All statistical analyses were performed using the SAS Statistical Package, version 9.4 (SAS Institute, Cary, NC, USA) and R version 3.5.1 (R Foundation for Statistical Computing, Vienna, Austria; ISBN 3–900,051–07-0, URL http://www.r-project.org) with the stats package 2.3. All statistics tests were two-tailed. CIs were considered significant when they did not include zero, and *p*-values < 0.05 were considered significant.

## Results

Overall, 639 patients were screened. After implementing the inclusion and exclusion criteria, 472 patients were enrolled in this study. The mean age of the patients at the time of assessment was 12.0 ± 7.0 years (Table 1, Fig. 1).

**Table 1:** Summary of patient data (*n* = 472)

Parameters	Values
Sex (male/female)^a^	302/170
GMFCS level (I/II/III/IV/V/not described)^a^	128/132/90/68/53/1
Involvement (bilateral/unilateral)^a^	394/78
Age at assessment^b^	12.0 ± 7.8

^a^Data are presented as number of patients

^b^Data are presented as mean ± standard deviation

*GMFCS* Gross Motor Function Classification System

The measurements of femoral anteversion and tibial torsion from 3D CT images showed good to excellent intra-observer and inter-observer reliabilities (Table 2). With age, external tibial torsion increased, while femoral anteversion decreased (Table 3). Although femoral anteversion decreased with age, the mean femoral anteversion was 41.0° (95% CI, 39.2–42.7) at skeletal maturity.

**Table 2:** Inter-observer and intra-observer reliability of the radiographic measurements

	Inter-observer reliability		Intra-observer reliability	
Measurement	ICC	95% CI	ICC	95% CI
Femoral anteversion	0.912	0.787–0.960	0.967	0.896–0.986
Tibial torsion	0.972	0.944–0.986	0.976	0.954–0.988

*ICC* Intraclass correlation coefficient, *CI* Confidence interval

**Table 3:** Estimation of femoral anteversion and tibial torsion by age in the linear mixed model

	< 4 years	5 years	6 years	7 years		8 years	9 years	10 years
Femoral anteversion	44.9	44.6	44.3	44.1		43.8	43.5	43.2
95% CI	45.5–45.3	44.1–45.1	43.8–44.9	43.4–44.7		43.0–44.6	42.6–44.4	42.3–44.2
Tibial torsion	13.7	14.0	14.3	14.6		14.9	15.2	15.5
95% CI	13.4–14.1	13.6–14.4	13.8–14.8	14.0–15.2		14.2–15.6	14.4–16.0	14.6–16.3
	11 years	12 years	13 years	14 years	15 years	16 years	17 years	> 18 years
Femoral anteversion	43.0	42.7	42.4	42.1	41.8	41.6	41.3	41.0
95% CI	41.9–44.0	41.5–43.8	41.1–43.7	40.7–43.5	40.4–43.3	40.0–43.1	39.6–42.9	39.2–42.7
Tibial torsion	15.7	16.0	16.3	16.6	16.9	17.2	17.5	17.8
95% CI	14.8–16.7	15.0–17.1	15.2–17.5	15.4–17.9	15.6–18.2	15.8–18.6	16.0–19.0	16.2–19.4

*CI* confidence interval

Statistically significant factors affecting external tibial torsion were age, GMFCS levels IV/V, hemiplegia, and femoral anteversion. With a 1-year increase in age, external tibial torsion increased by 0.29° (*p* < 0.0001). External tibial torsion was 4.30° greater in GMFCS levels IV and V than in GMFCS levels I and II (*p* < 0.0001). Both involved and uninvolved sides of hemiplegic patients were statistically significant factors affecting tibial torsion, with the involved side showing 2.63° (*p* = 0.0471) and the uninvolved side showing 3.87° (*p* = 0.0047) greater external tibial torsion in hemiplegic patients than in diplegic patients. Additionally, with 1° increase in femoral anteversion, external tibial torsion increased by 0.08° (*p* = 0.0057) (Table 4).

**Table 4:** Factors affecting tibial torsion in patients with cerebral palsy

		Estimate	95% CI	*p*-value
Intercept		12.55		
Age at assessment	years	**0.29**	0.20–0.38	**< 0.0001**
Sex (female)		1.06	−0.34–2.47	0.1380
Side (left)	(1/0)	5.69		
GMFCS level				
I/II	base			
III	(1/0)	−0.53	−2.40–1.33	0.5762
IV/V	(1/0)	**4.30**	2.61–6.00	**< 0.0001**
Diagnosis				
Diplegia (default)	base			
Hemiplegia (involved)		2.63	0.03–5.23	**0.0471**
Hemiplegia (uninvolved)		3.87	1.19–6.54	**0.0046**
FAV		0.08	0.02–0.14	0.0057

*CI* Confidence interval, *FAV* Femoral anteversion, *GMFCS* Gross Motor Function Classification System

In addition, factors affecting femoral anteversion were age, GMFCS level, and uninvolved limb in hemiplegia. With 1-year increase in age, femoral anteversion decreased by 0.28° (*p* < 0.0001). Femoral anteversion was 3.03° higher in GMFCS level III than in GMFCS levels I and II (*p* = 0.0036), whereas anteversion was 2.82° higher in GMFCS levels IV and V than in GMFCS levels I and II (*p* = 0.0029). The uninvolved side in hemiplegia was a statistically significant factor. The uninvolved limb showed 13.31° lower femoral anteversion in hemiplegic patients than in diplegic patients (*p* < 0.0001).

## Discussion

In this study, statistically significant factors affecting both external tibial torsion and femoral anteversion were age, GMFCS level, and hemiplegia. Femoral anteversion itself was a risk factor of increased external tibial torsion.

It is well-accepted knowledge that femoral anteversion improves with age in typically developing children (TDC) [14, 15]. Previous evidence from studies of hemiplegic CP patients proved a pattern contrary to that of TDC [16, 17]. This evidence was refuted in our study with CP patients; the analyzed patients tended to show a decrease in femoral anteversion as they aged, even in the affected limbs of hemiplegic patients. Although the overall pattern showed a decrease in femoral anteversion, the remaining deformity was great even at skeletal maturity, necessitating an interventional procedure to correct the deformity. Previous evidence regarding TDC has shown an increase in external tibial torsion as children age [15], yet only a few studies examined the rotational profiles of the tibia in patients with CP. Our study results showed that external tibial torsion in CP patients also followed the pattern seen in TDC, showing increase with age. This pattern may be a developmental change, or it may be a compensatory change to decreased femoral anteversion.

In tibial torsion and femoral anteversion, patients in GMFCS levels IV and V showed higher values than those in GMFCS levels I and II. Previous studies have shown that femoral anteversion and femoral neck-shaft angle tend to be higher in patients in GMFCS levels IV and V [3]. Our results are consistent with the findings of previous studies, probably because of increased spasticity, delay in motor development and weakness expressed in patients with an aggravated functional status. Our findings regarding hemiplegic patients are difficult to explain. There is no evidence as to why hemiplegic patients tended to show higher femoral anteversion than diplegic patients. Further study is required for a reasonable explanation of this phenomenon.

External tibial torsion tended to increase with increasing femoral anteversion. This may be a compensatory phenomenon to increase the femoral anteversion to maintain neutral foot progression. The relationship between pelvic external rotation as a result of increased femoral anteversion has been proven in a previous study [2]. Additionally, a study has shown that correction of increased femoral anteversion also corrects the external pelvic torsion [5, 18]. If external pelvic torsion is a short-term compensatory mechanism of increased femoral anteversion, it is our speculation that increased external tibial torsion may be a long-term consequence of increased femoral anteversion.

Excessive external tibial torsion causes torsional malalignment, which impairs the functional lever arm needed for adequate transfer of ground reaction force [1]. Missed external tibial torsion may also cause aggravation of out-toeing gait after correction of increased femoral anteversion. Tibial torsion shows lower validity between physical examination and CT than does femoral anteversion [7, 8]. Thus, it is challenging to evaluate the extent of external tibial torsion solely using physical examination, and an imaging study is crucial for evaluation. Therefore, in patients with increased external tibial torsion and risk factors, an imaging study is recommended to assess the severity of external tibial torsion.

It is crucial to mention the limitations of our study. First, the study had a retrospective design, and a uniform protocol was not implemented. In addition, sex, age, GMFCS level, and laterality at assessment could not be unified; however, a linear mixed model was implemented to overcome this limitation [10]. Second, selection bias must be addressed in our study. The examined patients had either clinically or physically suspected torsional malalignment, which is why they underwent torsional CT. Therefore, this study’s results may be applied to the general population. However, our goal was to address the presence of excessive tibial torsion in those expressing rotational gait problems and to determine the risk factors that contribute to this phenomenon. Thus, we believe that even though our results may not be representative of the general population, they are more relevant to the setting where we see our patients in the clinic. Third, this study is limited to evaluation of external tibial torsion. Evaluation of internal tibial torsion has not been done, which may foster future study. Fourth, although we have observed statistically significant risk factors of external tibial torsion, our results may not be clinically significant. However, this study is to set evidence for other clinicians that in patients with asserted risk factors, follow-up of changes in external tibial torsion should be performed along with femoral anteversion. Fifth, although the landmarks we have used for femoral anteversion and tibial torsion are set, the normal reference values for these two landmarks in TDCs are void. A future study may be conducted on setting the refence of femoral anteversion and tibial torsion in TDCs. Due to the cross-sectional nature of this study, the severity of external tibial torsion according to each age group could not be assessed. A future longitudinal analysis may be done for further analysis on external tibial torsion. Lastly, because we focused on external tibial torsion, an analysis on pes planovalgus, a foot deformity often co-exists with external tibial torsion, was excluded in this study. Further study is needed to discuss relationship between pes planovalgus and external tibial torsion.

## Conclusions

Older age, GMFCS levels IV/V, hemiplegia, and increased femoral anteversion are independent risk factors of increased external tibial torsion in patients with CP. Therefore, those with addressed risk factors of increased external tibial torsion, a careful surveillance of external tibial torsion is recommended.

## Data Availability

The data set supporting the conclusion of this article is available on request to the corresponding author.

## Abbreviations

CP: Cerebral palsy
GMFCS: Gross Motor Function Classification System
IRB: Institutional review board
CDW: Clinical data warehouse
HIMSS: Healthcare Information and Management Systems Society
3D: 3-dimensional
CT: Computed tomography
AIC: Akaike information criterion
BIC: Bayesian information criterion
ICCs: Intraclass correlation coefficients
CI: Confidence interval
TDC: Typically developing children

## References

[1] Theologis T (2013). Lever arm dysfunction in cerebral palsy gait. J Child Orthop.

[2] Rethlefsen SA, Kay RM (2013). Transverse plane gait problems in children with cerebral palsy. J Pediatr Orthop.

[3] Robin J, Graham HK, Selber P, Dobson F, Smith K, Baker R (2008). Proximal femoral geometry in cerebral palsy: a population-based cross-sectional study. J Bone Joint Surg British.

[4] Kwon DG, Lee SY, Kim TW, Chung CY, Lee KM, Sung KH (2013). Short-term effects of proximal femoral derotation osteotomy on kinematics in ambulatory patients with spastic diplegia. J Pediatr Orthop B.

[5] Ounpuu S, Solomito M, Bell K, Pierz K (2017). Long-term outcomes of external femoral derotation osteotomies in children with cerebral palsy. Gait Posture.

[6] Sung KH, Kwon SS, Chung CY, Lee KM, Cho GH, Park MS (2018). Long-term outcomes over 10 years after femoral derotation osteotomy in ambulatory children with cerebral palsy. Gait Posture.

[7] Chung CY, Lee KM, Park MS, Lee SH, Choi IH, Cho TJ (2010). Validity and reliability of measuring femoral anteversion and neck-shaft angle in patients with cerebral palsy. J Bone Joint Surg Am.

[8] Lee SH, Chung CY, Park MS, Choi IH, Cho TJ (2009). Tibial torsion in cerebral palsy: validity and reliability of measurement. Clin Orthop Relat Res.

[9] Dobson AJaB AG (2008). Introduction to generalized linear models.

[10] Kwon SS, Lee KM, Chung CY, Lee SY, Park MS. An introduction to the linear mixed model for Orthopaedic research. JBJS Rev. 2014;2(12). 10.2106/JBJS.RVW.N.00009 27490509

[11] Lee KM, Lee J, Chung CY, Ahn S, Sung KH, Kim TW (2012). Pitfalls and important issues in testing reliability using intraclass correlation coefficients in orthopaedic research. Clin Orthop Surg.

[12] Shrout PE, Fleiss JL (1979). Intraclass correlations: uses in assessing rater reliability. Psychol Bull.

[13] Bonett DG (2002). Sample size requirements for estimating intraclass correlations with desired precision. Stat Med.

[14] Jacquemier M, Glard Y, Pomero V, Viehweger E, Jouve JL, Bollini G (2008). Rotational profile of the lower limb in 1319 healthy children. Gait Posture.

[15] Staheli LT, Corbett M, Wyss C, King H (1985). Lower-extremity rotational problems in children. Normal values to guide management. J Bone Joint Surg Am.

[16] Staheli LT, Duncan WR, Schaefer E (1968). Growth alterations in the hemiplegic child. A study of femoral anteversion, neck-shaft angle, hip rotation, C.E. angle, limb length and circumference in 50 hemiplegic children. Clin Orthop Relat Res.

[17] Beals RK (1969). Developmental changes in the femur and acetabulum in spastic paraplegia and diplegia. Dev Med Child Neurol.

[18] Cimolin V, Piccinini L, Portinaro N, Turconi AC, Albonico S, Crivellini M (2011). The effects of femoral derotation osteotomy in cerebral palsy: a kinematic and kinetic study. Hip Int.

